# Enhanced natural killer cell anti-tumor activity with nanoparticles mediated ferroptosis and potential therapeutic application in prostate cancer

**DOI:** 10.1186/s12951-022-01635-y

**Published:** 2022-09-29

**Authors:** Kwang-Soo Kim, Bongseo Choi, Hyunjun Choi, Min Jun Ko, Dong-Hwan Kim, Dong-Hyun Kim

**Affiliations:** 1 Department of Radiology, Feinberg School of Medicine, Northwestern University, Chicago, IL 60611 USA; 2 Department of Biomedical Engineering, University of Illinois, Chicago, IL 60607 USA; 3 School of Chemical Engineering, Sungkyunkwan University (SKKU), Suwon, 16419 Korea; 4 Robert H. Lurie Comprehensive Cancer Center, Northwestern University, Chicago, IL 60611 USA; 5 Department of Biomedical Engineering, McCormick School of Engineering, Northwestern University, Evanston, IL 60208 USA

**Keywords:** NK cells, Ferroptosis, Cancer therapy, Nanomedicine, Prostate cancer

## Abstract

Ferroptosis provides an opportunity to overcome the cancer cell therapeutic resistance and modulate the immune system. Here an interaction between ferroptosis of cancer cells and natural killer (NK) cells was investigated with a clinical grade iron oxide nanoparticle (ferumoxytol) for potential synergistic anti-cancer effect of ferroptosis and NK cell therapy in prostate cancer. When ferumoxytol mediated ferroptosis of cancer cells was combined with NK cells, the NK cells’ cytotoxic function was increased. Observed ferroptosis mediated NK cell activation was also confirmed with IFN-γ secretion and lytic degranulation. Upregulation of ULBPs, which is one of the ligands for NK cell activating receptor NKG2D, was observed in the co-treatment of ferumoxytol mediated ferroptosis and NK cells. Additionally, HMGB1 and PD-L1 expression of cancer cells were observed in the treatment of ferroptosis + NK cells. Finally, in vivo therapeutic efficacy of ferumoxytol mediated ferroptosis and NK cell therapy was observed with significant tumor volume regression in a prostate cancer mice model. These results suggest that the NK cells’ function can be enhanced with ferumoxytol mediated ferroptosis.

**Supplementary Information:**

The online version contains supplementary material available at 10.1186/s12951-022-01635-y.

## Introduction

Ferroptosis has been increasingly studied as an alternative cancer cell killing pathway that can overcome the therapeutic-insensitivity and -resistance of cancer cells upon conventional apoptosis-based cancer therapies [1–6]. Emerging nanoparticles-mediated ferroptosis cancer therapies have been widely studied in recent years [7–9]. Iron based nanoparticles, upconversion nanoparticles, metal–organic framework nanoparticles, tumor microenvironment responsive nanoparticles, and so on are developed for promoting reactive oxygen species (ROS) accumulation and lipid peroxidation (LPO) in tumor cells [10]. Rationally designed combination nano-ferroptosis cancer therapy with conventional radiation or chemotherapy showed synergistic anti-cancer effect [11–13]. At the same time, as recently highlighted the promising clinical therapeutic efficacy of various immunotherapies, potential conjunction between ferroptosis and immune system has been paid much attention. Various ferroptosis related immunity research has been on-going. Up to now, it has revealed that ferroptotic cancer cells continuously release immunostimulatory damage-associated molecular pattern molecules (DAMPs) and activate the adaptive immune system within the tumor microenvironment (TME) to enhance anticancer effects [14, 15]. Further, Wang et al. [16] recently reported that IFN-γ secreted from cytotoxic CD8 + T cells induced the ferroptosis of cancer cells. The treatment of immune checkpoint inhibitors that activates CD8 + T cells further enhanced the ferroptosis of cancer cells. It is indicating that the cytotoxic immune cells can utilize the ferroptosis for killing cancer cells. Thus, the combination of ferroptosis and cytotoxic immune cell therapy could be a promising cancer therapeutic strategy in the future. However, there is little understanding of ferroptosis related immune response yet, so that further additional studies investigating the relationship between ferroptosis and various immune cells are warranted to develop ferroptosis based cancer immuno-therapies.

Among effective immune cell-cancer therapies, natural killer (NK) cell immuno-therapy has been accepted as a safe and effective tumor targeted therapeutic approach. Intrinsic NK cells’ cancer cell selective cytotoxic function controlled by the sensitive activation/inhibitory signaling balance has prompted their potential uses for the treatment of cancers. Clinical trials using autologous NK cells, allogeneic NK cells, modified NK cells have shown encouraging therapeutic efficacy [17–19]. However, the immune suppressive TME frequently inhibits NK cell activation and tumor infiltration of NK cells [20]. Additional efforts are required for developing highly efficient and broadly applicable NK cell-based immunotherapies in the clinic [21–25].

**Fig. 1:**
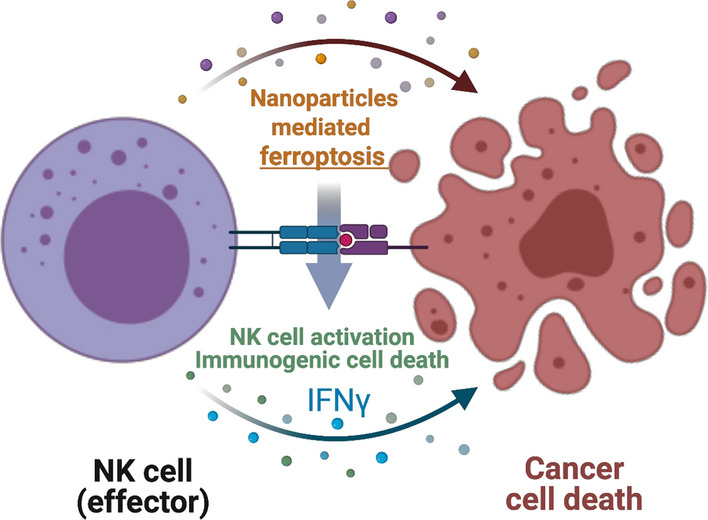
Schematic illustration of NK cell-based therapy with the ferumoxytol iron oxide nanoparticles mediated ferroptosis

In this study, we hypothesized that nanoparticles mediated ferroptosis induction of cancer cells enhances NK cells’ anti-cancer cytotoxic effect. Importantly, an interaction between ferroptosis of cancer cells and NK cells was investigated with clinical grade iron oxide nanoparticles (ferumoxytol) for potential synergistic anti-cancer effect of ferroptosis and NK cell therapy in prostate cancer cells (Fig. 1). Then, in vivo synergistic combination effect of ferumoxytol mediated ferroptosis and NK cell cancer therapy was evaluated in prostate cancer mice model.

## Results

**Fig. 2:**
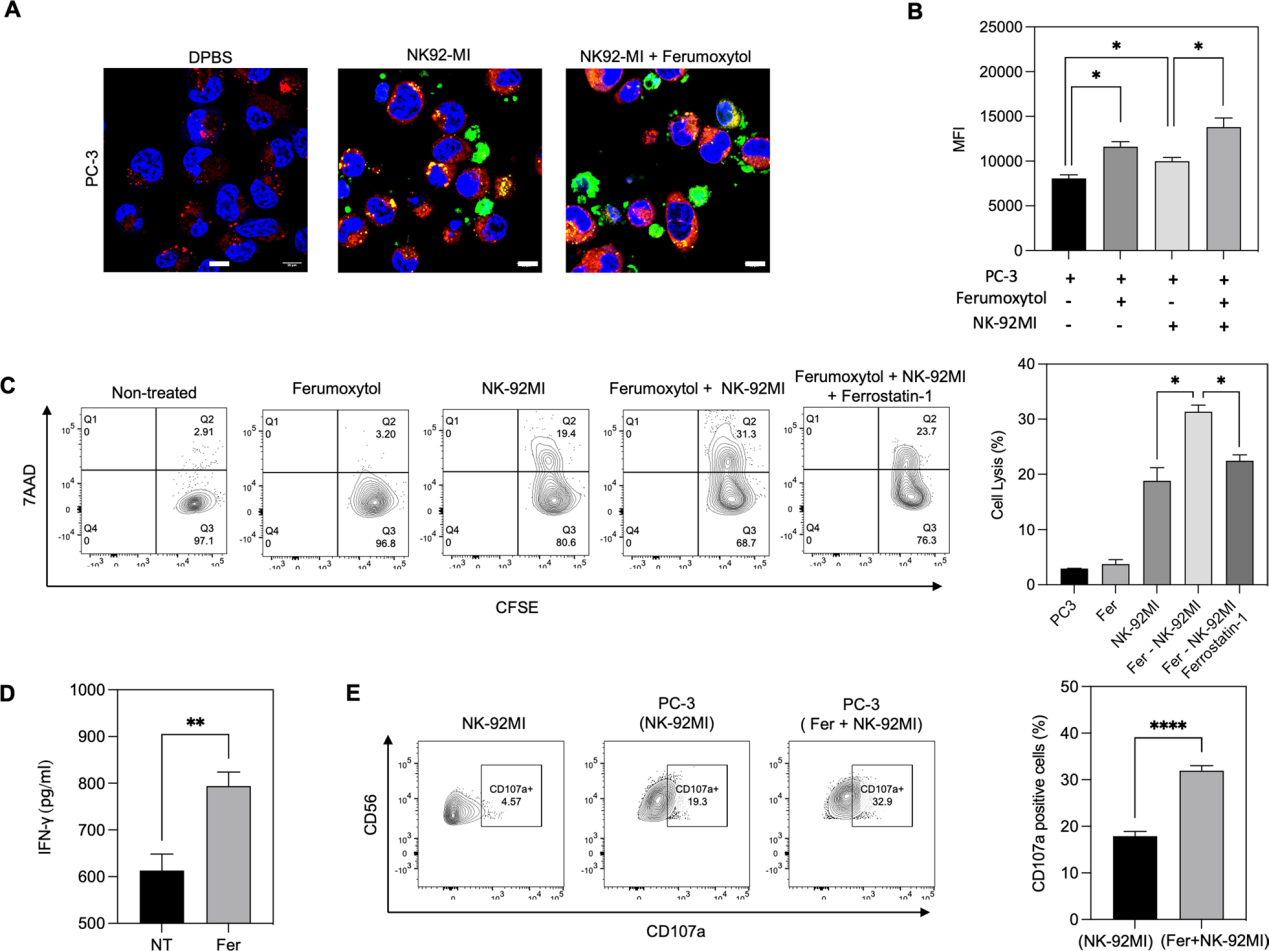
Ferumoxytol and NK cells synergistically induce ferroptosis in PC3 prostate cancer and the ferumoxytol mediated ferroptosis activates the cytotoxic function of NK cells. **A**. Confocal images of C11-BODIPY stained PC3 cells showed lipid peroxidation status. PC3 cells were treated with Ferumoxytol and co-cultured with NK-92MI cells (green). (scale bar = 20 µm) **B**. Fluorescent level of intracellular LPO in PC3 cells treated with each group was measured by flow cytometry. **C**. NK cell-mediated tumor cell killing effect of each treatment (ferumoxytol, NK-92MI, and ferumoxytol + NK-92MI) was determined by CFSE/7AAD assay. Ferrostatin-1 ferroptosis inhibitor was used to confirm the ferroptosis enhanced NK cell killing efficacy of the co-treatment ferumoxytol + NK-92MI. **D**. Interferon gamma secretion in only NK cell treatment and ferumoxytol + NK cell treatment. **E**. Degranulation of NK-92MI cells was determined by analysis of CD107a expression on NK cells. NK-92MI and PC3 cells were co-cultured at a 10:1 effector: target ratio and measured by flow cytometry. The data represent mean ± s.d. (n = 3) and statistical significance was analyzed by two-tailed Student’s t-tests. *P < 0.05, **P < 0.01, and ****P < 0.0001

Iron oxide nanoparticles have been discovered as an inducer of ferroptotic cell death in various kind of tumors [26–28]. Herein, ferumoxytol was used to evaluate the contribution of cancer cell ferroptosis in NK cell therapy. Ferumoxytol in our tested concentration range (~ 160 μg/mL) did not show significant cytotoxicity in both PC-3 cells and NK-92MI cells (Additional file 1: Fig. S1). 100 μg/mL of ferumoxytol was co-incubated with three different groups of human NK cells (NK-92MI), PC-3 human prostate cancer cells or NK-92MI + PC-3 human prostate cancer cells. Then, the ferroptosis induction of PC-3 cells in each group was measured with C11-BODIPY, a ferroptosis marker corresponding to the lipid ROS related with iron metabolism [29]. Confocal laser scanning microscope images and Western blot showed that ferumoxytol induced the ferroptosis of PC-3 cells (Fig. 2A and Additional file 1: Fig. S2). Interestingly, only NK cell treatment without ferumoxytol showed a similar level of ferroptosis with ferumoxytol. It is demonstrating that NK cells may induce the ferroptosis of cancer cells, as shown the ferroptosis induction of CD8 T cells, which are another effector lymphocytes, in a previously report [16]. Furthermore, the lipid ROS accumulation of PC-3 cells co-treated with ferumoxytol and NK cells was further increased compared to each mono-treatment of NK cells (Fig. 2A). Additional flow cytometry quantification of lipid peroxides (LPO) in cellular membranes [30] of treated PC-3 cells [30] confirmed the enhanced ferroptosis induction in the treatment of ferumoxytol + NK cell treatment. Co-treatment of ferumoxytol and NK cells showed the highest rate of PC-3 cell ferroptosis induction (Fig. 2B).

Next, cancer cell killing efficacy of combined ferroptosis and NK cell therapy was further investigated. Co-treatment of NK cell therapy and ferumoxytol mediated ferroptosis showed the highest cancer cell lysis rate synergistically increasing up to 31.3% compared to the mono- treatment of only NK cells or ferroptosis (Fig. 2C). NK cell treatment showed about 18% increment of PC-3 cell lysis and ferumoxytol mediated ferroptosis group showed only ~ 4% of cell lysis similar with spontaneous cell lysis in non-treated group. The improved cell killing effect of combined ferroptosis and NK cells was validated with ferroptosis inhibitor treated group. Ferroptosis inhibition of cancer cells using a ferroptosis inhibitor ferrostatin-1 resulted in significantly decreased cancer cell killing effect in NK cell + ferumoxytol (Fig. 2C). Boosted cytotoxic function of NK cells with ferumoxytol mediated ferroptosis might be attributed by ferroptosis mediated NK cell activation. A possible NK cell activation by the ferroptosis treatment was further measured with NK cell activation makers such as IFN-γ secretion and degranulation of NK cells after each treatment. In a comparison with NK cell treatment only, the co-treatment of NK cells and ferumoxytol mediated ferroptosis significantly increased 34% IFN-γ secretion (Fig. 2D). At the same time, CD107a surface expression of NK cell, which is a marker of NK cell degranulation, was also significantly enhanced in the co-treatment group of NK cells and ferumoxytol (Fig. 2E). While the basal expression of CD107a on only NK cells treatment was less than 5% and NK cells co-incubated with PC3 cells showed 19.3%, the NK cells + ferumoxytol mediated ferroptosis group significantly increased CD017a expression up to 32.9% (p < 0.0001) (Fig. 2E).

**Fig. 3:**
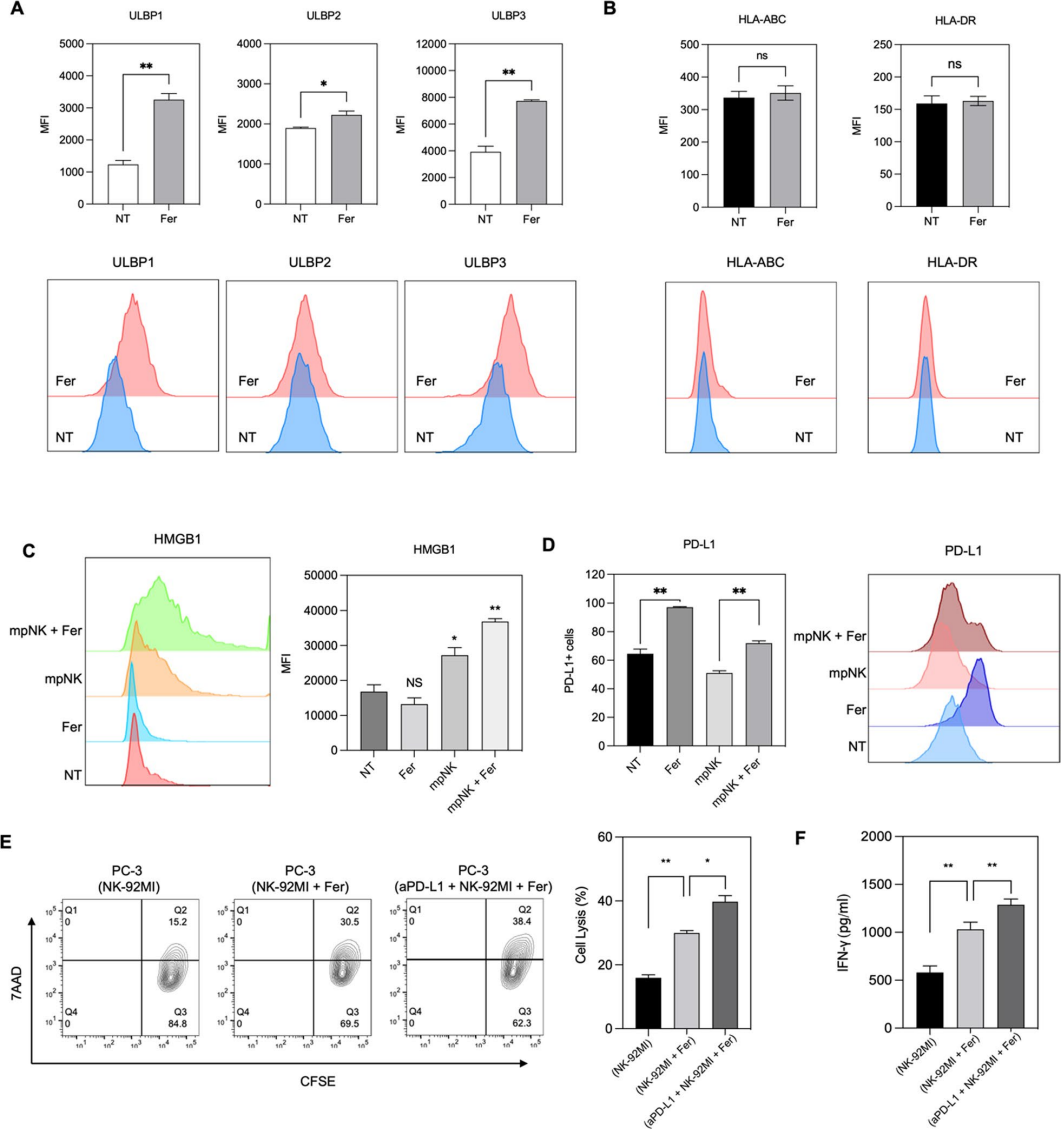
**A**. Expression of ULBPs on prostate cancer cells after Ferumoxytol treatment. Cancer cells were treated with ferumoxytol for 24 h and, expression of ULBPs was measured by flow cytometry. **B**. MHC class I and II expression on PC-3 prostate cancer cell were determined by flow cytometry **C**. HMGB1 expression of each treatment was determined by flow cytometry. Cancer cells were co-cultured with mouse primary NK cells with or without ferumoxytol at a 1:1 effector: target ratio, and HMGB1 expression was measured. **D**. Cell-surface expression of PD-L1 in response to ferumoxytol mediated ferroptosis, as determined by flow cytometry. **E**. NK cell tumor cell killing effect of each treatment (only NK cells, ferumoxytol + NK cells, and ferumoxytol + NK cells + aPD-L1) was determined by CFSE/7AAD assay. **F**. Interferon gamma secretion after each treatment (only NK cells, ferumoxytol + NK cells, and ferumoxytol + NK cells + aPD-L1). The data represent mean ± s.d. (n = 3) and statistical significance was analyzed by two-tailed Student’s t-tests. *P < 0.05, **P < 0.01, ***P < 0.001, ****P < 0.0001

Resulting augmented NK cell cytolytic function by the ferumoxytol mediated ferroptosis suggests ferroptosis-boosted NK cell activation against the cancer cells. Direct potential pathways of ferroptosis related NK cell activation have not been studied much yet. Here, we hypothesized that the ferumoxytol mediated ferroptosis of cancer cells upregulated NK cell activating surface molecules, resulting in the cytotoxic effect of NK cells. As considering ROS stress during the ferroptosis process, we tested possible ferroptosis mediated upregulation of stress-inducible cell surface proteins, which can be recognized by natural killer group 2D (NKG2D) expressed on the membrane of NK cells. NK cell activation signal for the cytotoxic effect is involving an activation receptor such as NKG2D. The activation receptor is mainly binding with stress-inducible molecules UL16 binding proteins (ULBPs) and MHC class I chain-related protein A and B (MICA/B) [31, 32]. The activation receptor/ligand interaction is a decisive factor for the generation of functional NK cells that is selectively attacking cancer cells [21, 33]. Thus, the ferroptosis mediated expression of stress inducible ULBPs and MICA/B ligands was measured with a flowcytometry analysis after the ferumoxytol mediated ferroptosis induction of PC-3 cells. We found that all ULBP family members (ULBP1, ULBP2 and ULBP3) are upregulated with the ferumoxytol mediated ferroptosis (Fig. 3A). However, MICA/B was not significantly affected by the ferroptosis induction of PC-3 cells (Fig. 3B). Although detail pathways of the synergistic effect of ferroptosis and NK cell therapy are not still clear yet, upregulated ULBP family ligand proteins interacting with NKG2D are intimately associated with the enhanced the cytotoxic efficacy of NK cells upon the addition of ferumoxytol mediated ferroptosis.

Recent reports also reveal an opportunity of ferroptosis for combining with cancer immunotherapies based on ferroptosis related specific immunity [9, 34, 35]. Here a possible immunogenic cell death of NK cell therapy + ferumoxytol mediated ferroptosis was investigated with upregulated high mobility group box 1 (HMGB1) expression of PC-3 cells (Fig. 3C). HMGB1 expression was synergistically enhanced in the co-treatment of NK cells + ferumoxytol while only ferumoxytol (100 μg/mL) or NK cells were not enough to show the significant increase of HMGB1 expression. However, at the same time, flow cytometry analysis revealed that the NK cells + ferumoxytol mediated ferroptosis treatment significantly upregulated PD-L1 expression, which was about 18% higher compared to non-treated or only NK cell treatment (Fig. 3D). In summary, ferumoxytol induced ferroptosis enhanced NKG2D ligands (ULBP) in tumor cells. Then, upregulated NKG2D ligands in tumor cells activated NK cells. Subsequently, the activated NK cells released an increased amount of IFN-γ. The enhanced IFN-γ in the treatment of NK cells + ferumoxytol mediated ferroptosis induced the PD-L1 upregulation of cancer cells [36].

Observed ferroptosis mediated PD-L1 expression of cancer cells might be challenging to further improve the effective cancer cell killing of NK cells + ferroptosis [37]. Since NK cells are expressing PD-1 as well as innate lymphoid cells, the expressed immune checkpoint inhibitors of cancer cells are an important consideration to achieve an enhanced synergistic anti-cancer effect of NK cell therapy with ferroptosis. aPD-L1 immune checkpoint inhibitors can be suggested as a potential option to enhance the NK cell engagement to cancer cells during the NK cells + ferumoxytol mediated ferroptosis treatment. Here we tested if the additional aPD-L1 treatment in NK cell + ferroptosis enhanced the cancer cell killing effect of combined NK cells and ferroptosis. When aPD-L1 immunotherapy was added in the treatment of NK cell + ferumoxytol mediated ferroptosis, significantly enhanced the cancer cell lysis effect (about 38.4%) was observed (Fig. 3E). Only NK cells or NK cells + ferumoxytol treatment group showed 15.2% and 30.5%, respectively (Fig. 3E). IFN-γ secretion was also significantly increased upon the addition of aPD-L1 compared to only NK cells or NK + ferumoxytol treated groups (Fig. 3F). As confirmed in confocal images (Additional file 1: Fig. S3), aPD-L1 assisted the engagement of NK cells to cancer cells. Subsequently, the cancer cell killing effect of NK cells + ferumoxytol mediated ferroptosis was further enhanced by the aPD-L1 treatment.

**Fig. 4:**
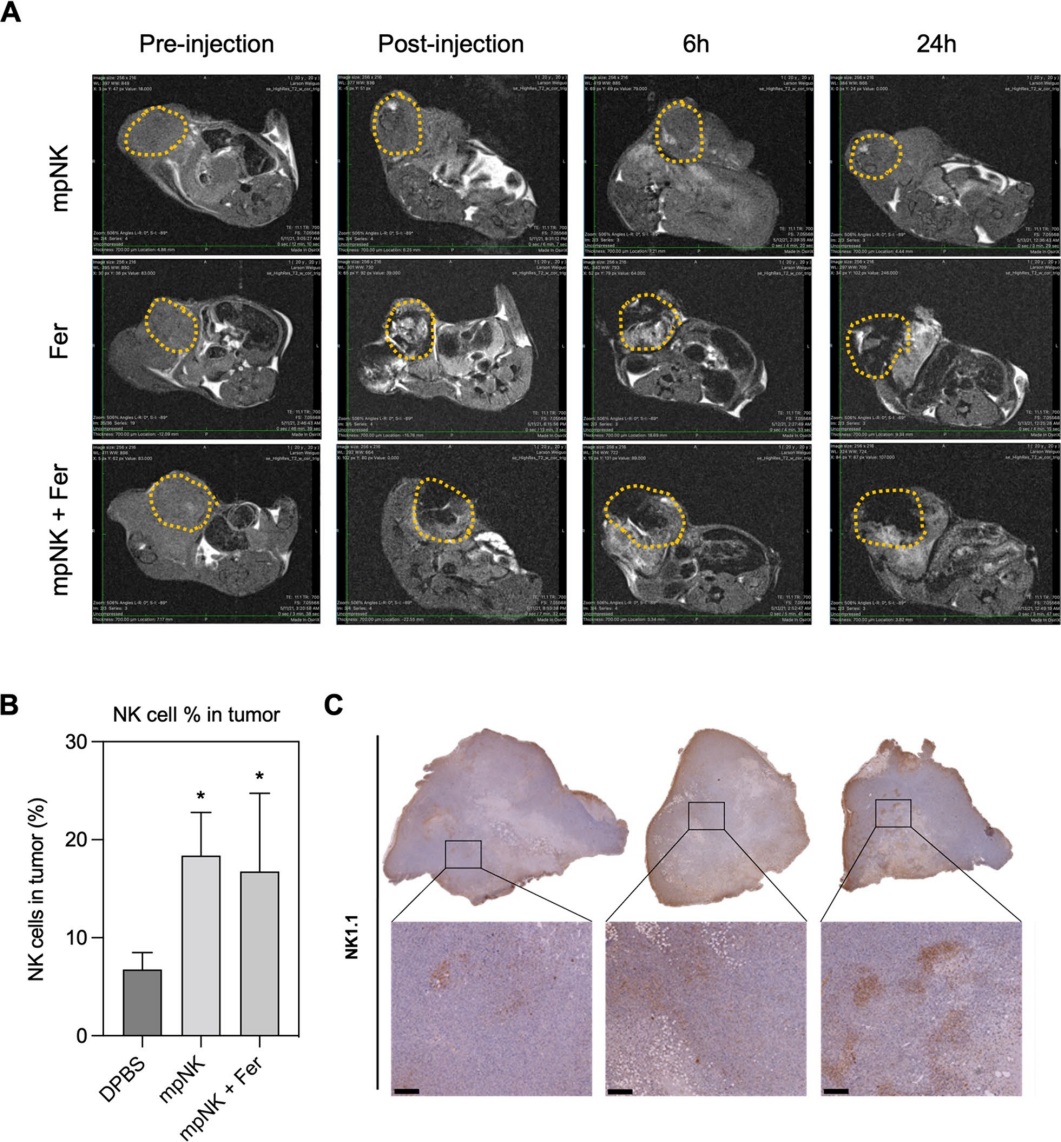
**A**. MRI T2 images of tumors treated with intra-tumoral injection of samples (only NK cells, ferumoxytol, and ferumoxytol + NK cells. **B**. Flowcytometry quantification of NK cells in tumors treated with only DPBS, NK cells, or ferumoxytol + NK cells. **C**. Representative immunohistochemical images of NK1.1 for each experimental group. (scale bar = 100 µm)

Next, for potential in vivo application of the combination NK cells and ferroptosis, the administration route of the combination therapeutic should be a critical consideration due the immune suppressive TME. Image guided percutaneous intra-tumoral injection of the treatment might be beneficial to localize ferroptosis and enhance the NK cell homing/intra-tumoral infiltration for solid tumors such as prostate cancer [21, 38]. Prior to in vivo therapeutic efficacy test of ferroptosis mediated enhancement of NK cell therapy, the feasibility of intra-tumoral injection of NK cells + ferumoxytol was investigated in TrampC1 prostate cancer mice models. When the NK cells and ferumoxytol was applied to the tumor, the injection site and distribution of the samples including MRI visible ferumoxytol was readily located with drawn ROIs using low MRI T2 signal intensity in the tumor (Fig. 4A). MRI T2 contrast was maintained in the tumor region during 24 h post-treatment. Flow cytometry data quantified the number of intra-tumoral NK cells at 4-day post injection. About 3-folds higher number of intra-tumoral NK cells was observed in the groups of intratumorally injected NK cells and NK cell + ferumoxytol compared to non-treated control tumor group (Fig. 4B). NK1.1 staining of tumor tissues also showed the higher positive signal of intra-tumoral NK cells in the intratumorally injected NK cell groups (Fig. 4C). MR image-guided intra-tumoral local infusion of NK cells might be an effective option for localizing NK cell dosage and overcoming various TME barriers impeding NK cell trafficking/infiltration into tumors [39, 40].

**Fig. 5:**
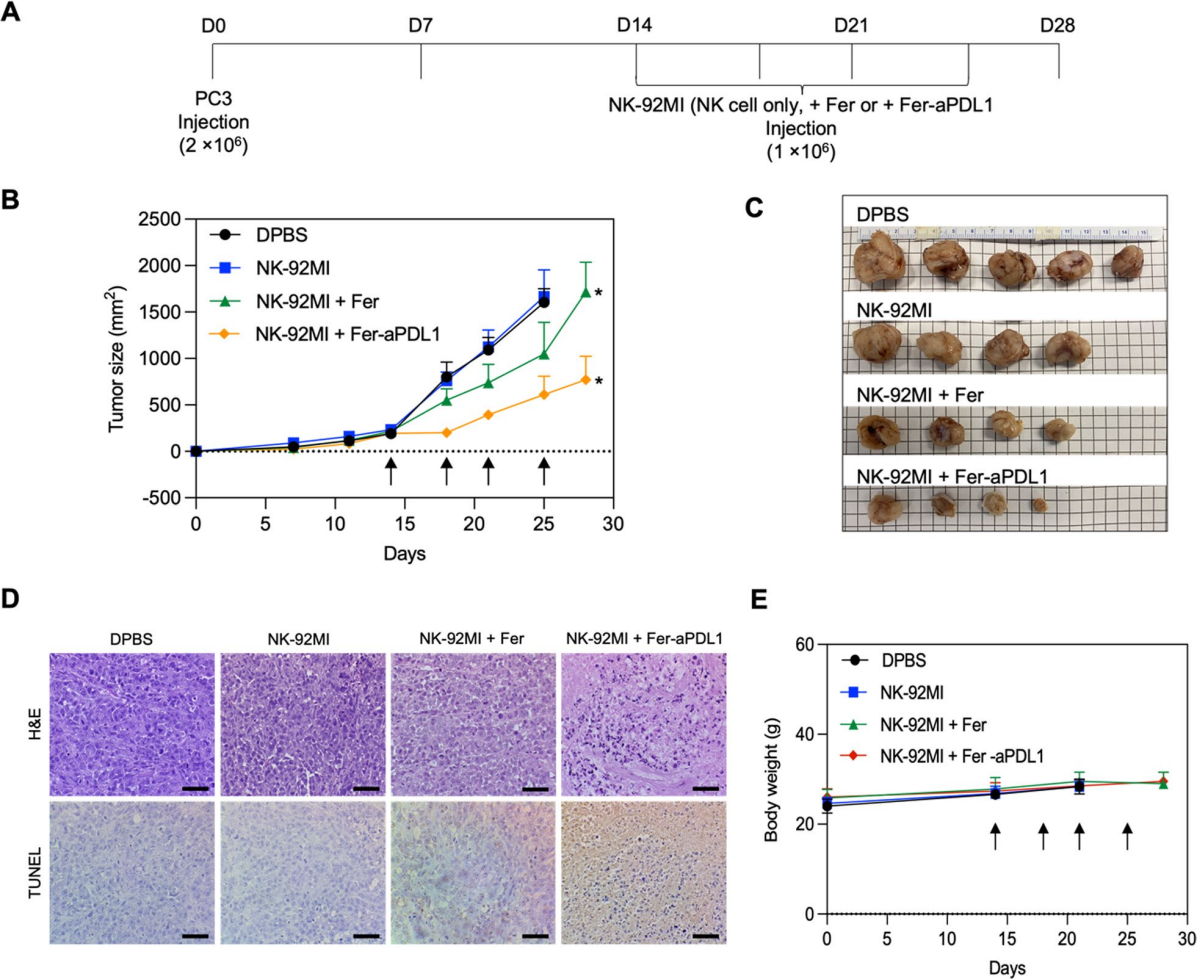
Direct anti-cancer activity of combination treatment of NK cells and ferumoxytol in the PC-3 prostate cancer mice model. **A**. Experimental design of in vivo direct anti-cancer effects. **B**. Tumor growth curve of each group of treatment. The 6–8-week-old mice were randomly divided into 4 groups, DPBS, NK-92MI, NK-92MI + Fer and NK-92MI + Fer-aPDL1 group. NK cells were intratumorally injected into the PC3 tumors 4 times on days of 14, 18, 21 and 25. **C**. Representative images of tumors extracted after the experiment. **D**. Representative H&E images and TUNEL images of each treatment group (scale bar = 50 µm). **E**. Mean change of body weight of mice during the experiment for each group. The data represent mean ± s.d. (n = 4, 5) and statistical significance was analyzed by two-tailed Student’s t-tests. *P < 0.05, **P < 0.01, ***P < 0.001, ****P < 0.0001

To evaluate direct therapeutic efficacy of intra-tumoral delivered NK cell therapy, PC-3 xenograft mice model was generated in athymic nude mice (male, 6–8 week). PC-3 mice bearing about 100 mm^3^ tumor were divided into 4 groups of non-treated (DPBS), NK cell therapy, NK cell + ferumoxytol, and NK cell + ferumoxytol + aPD-L1. All groups were injected 4 times in 2 weeks (twice a week), and the therapeutic response was measured with tumor volume changes and histology analysis (Fig. 5A). The tumor growth of NK-92MI treated group was relatively fast to reach over 2000 mm^3^ of tumor volume at 25-day post treatment, resulting in the sacrifice of mice. The tumor growth rate of NK-92MI treated group was similar with non-treated control tumors. However, when ferumoxytol mediated ferroptosis was combined with NK-92MI cell injection, the tumor growth rate of NK-92MI + ferumoxytol group was significantly suppressed by 38.1% (Fig. 5B, C, p < 0.05 and Additional file 1: Fig. S4). As demonstrated in vitro results, the addition of aPD-L1 treatment further enhanced the suppression of tumor growth. Enhanced cancer cell death in tumor tissues treated with groups of NK cell + Ferumoxytol and NK cell + Ferumoxytol + aPD-L1 was confirmed in H&E and TUNEL histology images (Fig. 5D). Additionally, there was no significant loss of body weight in all intratumorally injected groups, indicating low systemic toxicity of those combination treatment (Fig. 5E).

**Fig. 6:**
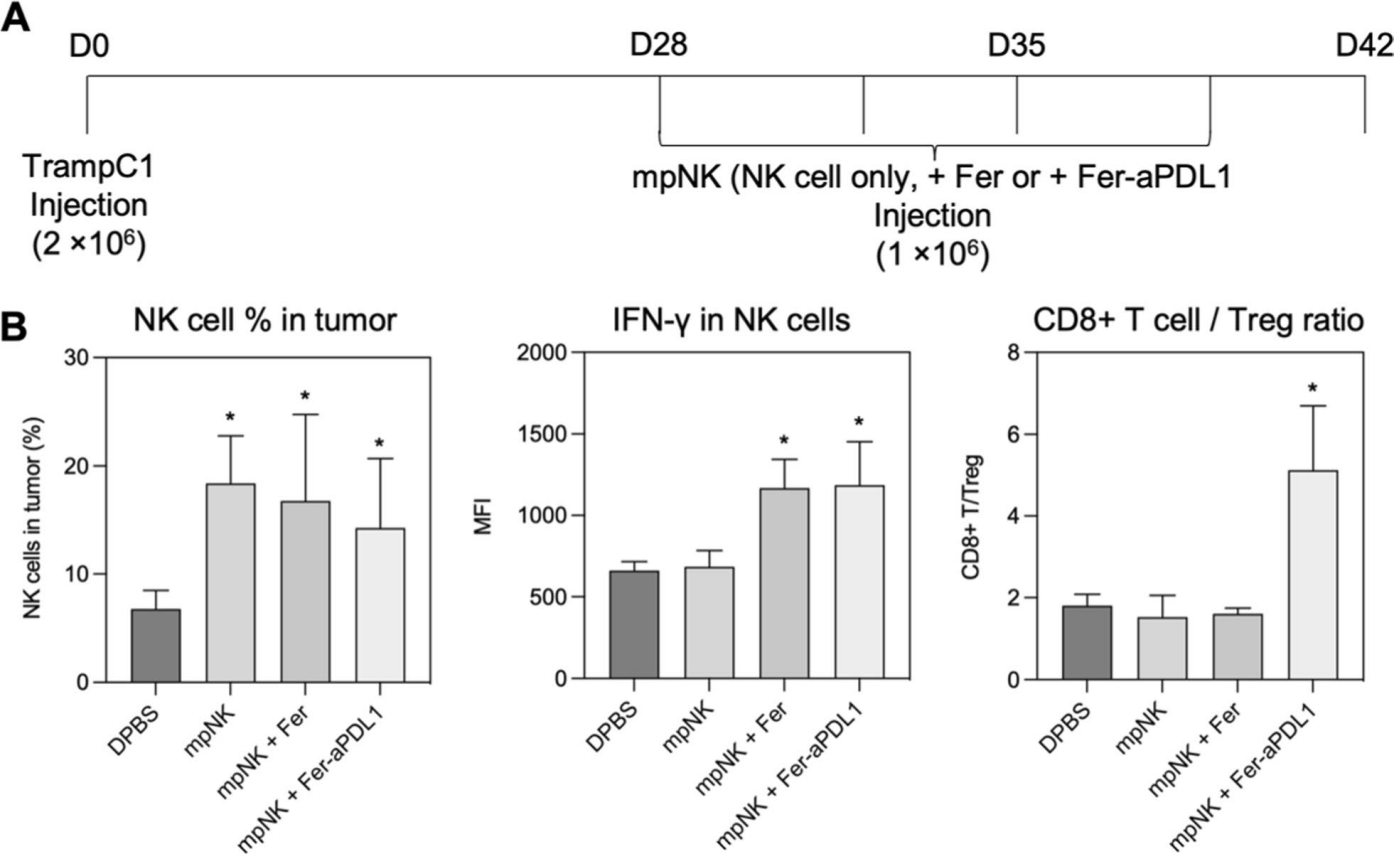
Remodeling of tumor immune environment in responses of NK cell and ferumoxytol mediated ferroptosis combined therapy. **A**. Experimental design of in vivo immune remodeling effect in syngeneic mice model. The 6–8-week-old male C57BL/6 mice were randomly divided into 4 groups, DPBS, mpNK, mpNK + Fer and mpNK + Fer-aPDL1 group. TrampC1 bearing C57BL/6 mice were injected NK cells intratumorally 4 times on days of 28, 32, 35 and 39. **B**. Flow cytometry analysis of the percentage of NK cells in the tumors, the amount of NK cells containing IFN-γ and the ratio of CD8 + T cell/ regulatory T cell in the tumors after indicated treatment. The data represent mean ± s.d. (n = 4) and statistical significance was analyzed by two-tailed Student’s t-tests. *P < 0.05, **P < 0.01, ***P < 0.001, ****P < 0.0001

Finally, we tested immune response of each treatment in a syngeneic mouse model. C57BL/6 mice (male, 6–8 week) were adapted to evaluate immune profiles after each treatment of DPBS, NK cells, NK cells + Ferumoxytol, and NK cells + Ferumoxytol + aPD-L1. We used TrampC1 mouse prostate cancer cell line, which is syngeneic with C57BL/6 hosts, and mouse primary NK cells isolated from C57BL/6 splenocytes. When tumors are reached to about 100 mm^3^ volume, DPBS, mouse primary NK cells (mpNK), mpNK + Ferumoxytol, and mpNK + Fer + aPD-L1 were intratumorally injected 4 times in 2 weeks with MR images, respectively (Fig. 6A). The production of IFN-γ, which is indicator of the activity of NK cells, was significantly upregulated in the groups of NK cell + Ferumoxytol and NK cell + Ferumoxytol + aPD-L1 compared to control or only NK cell treated groups (Fig. 6B). As shown aPD-L1 mediated NK cell activation property (Figs. 2D, E and  3), NK cells + Ferumoxytol + aPD-L1 treatment showed significant enhancement of T cell activation compared to other groups (Fig. 6B). The addition of aPD-L1 therapy changed CD8 + T cell profile. CD8 + cytotoxic T cell and regulatory T cell ratio was significantly increased by the addition of aPD-L1 (NK cells + Ferumoxytol + aPD-L1).

## Discussion

Conventional cancer therapies including surgery, chemotherapy and radiotherapies have not shown significant therapeutic outcomes with cancer cell therapeutic-resistance and -tolerance. Emerging cancer immunotherapy as the most promising future cancer medicine is also demonstrating limited therapeutic efficacy. Ferroptosis is a newly identified type of cell death dependent on lipid peroxidation, which differs from classical programmed cell death in terms of morphology, physiology, and biochemistry. Recent studies have suggested that ferroptosis could overcome the cancer cell therapeutic resistance and modulate the immune system, describing the potential physiological functions of ferroptosis in tumor suppression and immune surveillance. A representative study demonstrated that CD8 + T cells increased the ferroptosis through IFN-γ, and the ferroptosis is directly sensitizing cancer cells to various immunotherapies. Combination of ferroptosis, immune cell therapy, and immune checkpoint inhibitor therapy would be an effective strategy to improve the limited cancer therapeutic outcomes. Here NK cell therapy was tested as a potential combination with ferroptosis mechanism. A rising number of approaches are being actively exploited to boost NK cell-based anti-tumor immune functions. Immune-stimulatory molecules can selectively enhance NK cell activity while maintaining their in vivo survival and proliferation (e.g., cytokines), or mediate NK cell cytotoxicity by ADCC (e.g., antibodies). With the advances in ex vivo expansion/activation technology, as well as new approaches to combination therapy with NK cells, adoptive transfer of NK cells holds promises to become powerful weapons in the fight against cancers. Functional cytokines (IL-2, 15, 18, 21, IFN-γ) and conventional chemo-drugs (paclitaxel, docetaxel, lenalidomide) combinations have been suggested to improve NK cell function in solid tumors [41–43]. Regardless of these potential strategies, the clinical outcomes of NK cell therapy have been still moderate in solid tumors [44, 45]. One of emerging direction of NK cell therapy has been the combination NK cell therapy with additional therapeutics that can modulate TME [21]. Finding an optimal combination treatment with NK cell therapy is of great interest for the enhanced NK cell cancer therapy.

**Fig. 7:**
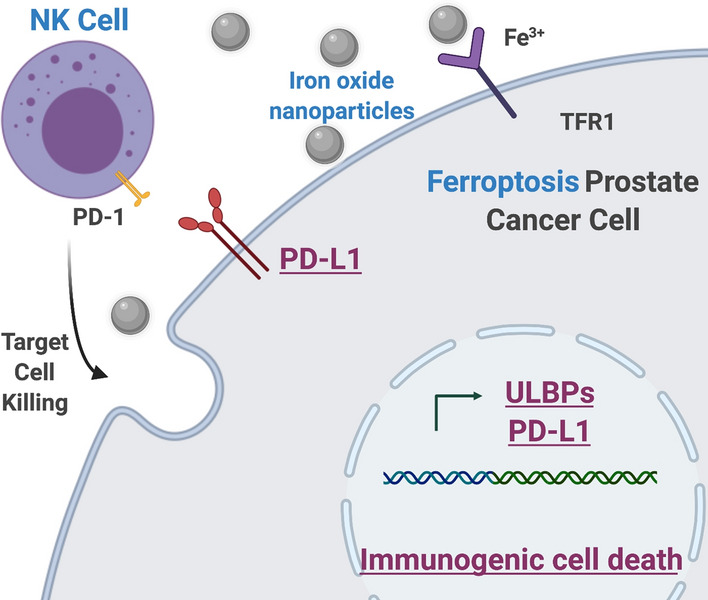
Suggested mechanism of NK cell therapy with the ferumoxytol mediated ferroptosis. Imbalance of iron metabolism in the prostate cancer cells induces ferroptosis. Resulting ferroptosis induces expression of ULBPs and PD-L1 with immunogenic cell death. Nanoparticles mediated ferroptosis and NK cell therapy synergistically enhances the cancer cell killing effect in prostate cancer

Here the potential relationship between ferroptosis and NK cells was investigated with ferumoxytol mediated ferroptosis and following NK cell activation (Fig. 7). Ferumoxytol is an FDA approved iron oxide nanoparticle, which is showing a low toxicity in normal tissues. Combination of ferumoxytol iron oxide nanoparticles with image guided delivery will enhance the tumor targeted ferroptosis induction [9]. Ferumoxytol iron agents directly induced ferroptotic LPO accumulation in prostate cancer cells having a high ferroptosis susceptibility with a defective iron metabolism and iron channel [46]. Since the cell viability wasn’t affected significantly, it could be considered as early ferroptosis (Additional file 1: Fig. S2). Ferumoxytol showed anti-cancer activity by regulating iron metabolism in cancer cells. Especially macrophages and T cells are involved in the mechanism [26, 47]. However, the interaction between NK cell and ferumoxytol mediated ferroptosis was still unknown. Our results demonstrated that the NK cells’ cytotoxic function was increased with the ferroptosis of cancer cells. The NK cell activation was confirmed with the IFN-γ secretion and lytic degranulation. It is note worth that ULBPs ligands for NK cell activation were significantly upregulated in the ferroptotic cancer cells (Fig. 7). This is a crucial data that describes the synergistic anti-cancer effect of ferroptosis and NK cells. Demonstrated HMGB1 and PD-L1 expression after the treatment of ferumoxytol mediated ferroptosis and NK cells provided additional important clues for the combination with aPD-L1 immune checkpoint inhibitor immunotherapy (Fig. 7). Since NK cells are expressing PD-1 as well as innate lymphoid cells, the addition of aPD-L1 indeed boosted the cytotoxic function of NK cells. Those enhanced anti-cancer effect of NK cell therapy with ferroptosis and aPD-L1 was confirmed with a significant tumor volume regression in prostate cancer mice model. Additional investigation of molecular features linked to the response to ferumoxytol mediated ferroptosis is required in a connection with the synergistic NK cell therapeutic effect. Further immune characterization such as CD8 + T cell activation and anti-cancer immunity would be the next step to translate this ferroptosis enhanced NK cell therapy for an effective NK cell therapy approach. Considering potential toxicity and low efficacy of systemically applied ferroptosis and NK cell therapies[21, 48–50], the development of emerging image guided local ferroptosis and NK cell therapies is needed [7, 21]. Additionally, although ferroptosis involved with iron, lipids and ROS are related with the very common metabolic pathways of cancer cells, their roles and contribution in the cancer treatment have remained unclear. More understanding the immunogenicity of ferroptosis would provide us new ideas in designing ferroptosis-based immuno-therapies. Nevertheless, the NK cell therapy can be improved with the ferumoxytol mediated ferroptosis and these results are providing an important potential of ferroptosis mediated immunotherapies.

## Materials and Methods

### Cell culture

NK-92MI (human NK cell line) and PC3 (human prostate cancer cell line) TrampC1 (mouse prostate cancer cell line) were purchased from the American Type Culture Collection (ATCC). NK-92MI cells were cultured in Minimum Essential Medium alpha (Invitrogen) supplemented with 2 mM L-glutamine (Invitrogen), 1.5 g/L sodium bicarbonate (Sigma-Aldrich), 0.2 mM inositol (Sigma-Aldrich), 0.1 mM 2-mercaptoethanol (Invitrogen), 0.02 mM folic acid (Sigma-Aldrich), 12.5% fetal bovine serum, and 1% penicillin/streptomycin. PC3 cells were grown in standard RPMI medium supplemented with 10% fetal bovine serum and 1% penicillin/streptomycin (Invitrogen). TrampC1 cells were cultured in Dulbecco's modified Eagle's medium with 4 mM L-glutamine adjusted to contain 1.5 g/L sodium bicarbonate and 4.5 g/L glucose supplemented with 0.005 mg/ml bovine insulin and 10 nM dehydroisoandrosterone, 90%; fetal bovine serum, 5%; Nu-Serum IV, 5%. Mouse primary NK cells were purified from spleen and characterized by flow cytometry analysis.

### Cytotoxicity assay of NK cells

1 × 10^5^ cells of PC-3 were pre-stained with Cell Trace CFSE (Invitrogen) and then co-cultured with NK-92MI cells at 10:1 effector: target (E: T) ratio. After 4 h of co-incubation, cells were harvested and stained with 7-AAD (Invitrogen) for the discrimination of live and dead cells. To assess the degranulation of NK cells, co-cultured cells were stained with anti-human CD107a-APC (BD Biosciences) for 1 h at 4 °C, then analyzed on a flow cytometer (BD Bioscience).

### Cell viability test

Cell viability was quantified by the colorimetric assay by using CCK-8 reagents (Dojindo Inc.). A total of 1 × 10^4^ cells in 100 μL of complete media were plated in each well of a 96-well cell culture plate. After overnight cultivation, 100 μL of media, containing 0 to 160 μg/mL concentration of ferumoxytol, was added to each well and incubated for 72 h. Then, CCK-8 reagents were treated by the manufacturer’s instructions. After 4 h of additional incubation, the absorbance of the plate was measured by spectrophotometer (BioTek Inc.) at 450 nm (OD).

### Flow cytometry

NK-92MI cells were treated with ferumoxytol for 24 h, then washed with cold DPBS and FACS buffer (BD Bioscience). Cells resuspended in FACS buffer were blocked with Fc blocker (BD Bioscience) for 15 min at 4 °C, and then incubated with dye-conjugated antibodies for 1 h at 4 °C. Antibody-labeled samples were fixed with 4% paraformaldehyde. Antibodies used for flow cytometry were purchased from Biolegend. Flow cytometry analysis was performed using BD fortessa flow cytometer (BD Bioscience).

### Enzyme-linked immunosorbent assay

1 × 10^4^ target cells were cocultured with effector cells at an effector cell: target cell (E: T) ratio of 10: 1 in round-bottom 96-well culture plates for 4 h. Cell-free supernatants were assayed for cytokine secretion by enzyme-linked immunosorbent assay (ELISA) kits according to the manufacturer’s protocol. Human and mouse IFN-γ ELISA kits were purchased from BioLegend.

### Confocal laser scanning microscopy

For confocal fluorescence microscopy, target tumor cells were plated at least 16 h before use on confocal dish (MatTek). After attached target cells, CFSE stained NK92-MI cells were added and co-incubated for 4 h. The co-incubated cells were treated with Hoechst 33,342 for visualize nucleus and fixed with 4% paraformaldehyde. Images were taken with a Nikon A1R spectral confocal laser scanning system on an inverted microscope (Nikon) with an argon-krypton laser as excitation source. The green and red fluorescence of C11-BODIPY581/591 was acquired simultaneously using double wavelength excitation (laserlines 488 and 568 nm) and detection (emission bandpass filters 530/30 and 590/30).

### Western blot analysis

PC-3 cells were seeded in the 8 cm culture dishes and cultured at 5% CO_2_, 37 °C overnight. The cells were treated with various amounts of ferumoxytol (Fer (0 μg), Fer (50 μg), Fer (100 μg), and Fer (200 μg)), respectively, for 12 h. No treatment of PC-3 cells was used as control. The cell lysates were collected and analyzed by Western blotting according to the manufacturer’s instructions. Antibodies were used listed as below: GPX4 Rabbit mAb, Cell Signaling, catalog number: 59735S, dilution 0.1–0.5 µg/mL; SLC7A11 rabbit mAb, Cell Signaling, catalog number: 12691S, dilution 0.1–0.5 µg/mL; Goat Anti-Rabbit IgG H&L (HRP), Abcam, catalog number: ab205718, dilution 1:2000; β-Actin (8H10D10) Mouse mAb (HRP Conjugate), cell signaling, catalog number:12262, dilution 1:1000.

### Intra-tumoral injection of NK cells

All experimental procedures were approved and reviewed by the Institutional Animal Care and Use Committee of Northwestern University (IS00002377). A total of 2 × 10^6^ Tramp C1 cells in 100 μL of PBS were subcutaneously injected into the right flank of C57BL/6 mice (male, 8–10 weeks old). After three weeks, tumor bearing mice with 100 to 150 mm^3^ volume were treated with NK cells and Ferumoxytol by intra-tumoral injection. MRI studies were performed using a Bruker 7.0 T ClinScan (Bruker BioSpin, Ettlingen, Germany) high-field small animal MRI system with a commercial mouse coil (Bruker Biospin).

### In vivo therapeutic efficacy

All experimental procedures were approved and reviewed by the Institutional Animal Care and Use Committee of Northwestern University. PC3 cells (2 × 10^6^ cells per mouse) in DPBS were injected into nude mice (male, 8–10 weeks old). After two weeks, tumor bearing mice were treated randomly with NK-92MI cells and Ferumoxytol. All mice were treated total 4 times in every 2–3 days. Tumor sizes were traced twice a week for 4 weeks.

### Prostate cancer mouse model and T cell Characterization

A total of 1 × 10^6^ Tramp C1 cells in 100 μL of PBS were subcutaneously injected into the right flank of C57BL/6 mice (male, 10 weeks old). After three weeks, tumor bearing mice with 100 to 150 mm^3^ volume were collected and treated with NK cells and Ferumoxytol by intra-tumoral injection. Tumor sizes were traced twice a week for 3 weeks. Then, mice were terminated, and T cell distributions (CD3, CD4, CD8, IFN-γ, CD25, FoxP3, CD45, IL17A, BD Biosciences) of tumor and spleen were analyzed by flow cytometry. Gating strategy is showing in Additional file 1: Fig S4.

### Histology and Immunohistochemistry

To assess the distribution of NK cells, tumors were harvested from the mice and fixed for 24 h in 4% paraformaldehyde. The tissue Sects. (5 μm) were stained with hematoxylin and eosin (H&E). Immunohistological analysis was performed using a terminal transferase dUTP nick-end labeling (TUNEL) assay (Promega) or antibodies including anti-mouse NK1.1 (Thermo). All slides were scanned using Hamamatsu K.K. Nanozoomer 2.0 and analyzed by ImageJ software to determine the positive cells per field of view.

### Statistical analysis

All statistical analyses were performed using the GraphPad Prism 8.0 software (GraphPad Software, Inc., La Jolla, CA, USA). Graphical data are presented as means ± standard deviation. Statistical significance of differences between two groups was determined using Student's unpaired t-test. P-values less than 0.05 were considered statistically significant.

## Supplementary Information

**Additional file 1**: **Figure S1**. Ferumoxytol concentration dependent Cell viability changes of PC3 and NK92-MI. Ferumoxytol (0–160 μg/mL concentration) was added to each well and incubated for 72 h. Then, CCK-8 reagents were treated by the manufacturer’s instructions. **Figure S2**. Western blot analysis of PC-3 cells treated with various amounts of ferumoxytol (Fer (0 μg), Fer (50 μg), Fer (100 μg), and Fer (200 μg)) and quantification of relative bands of western blotting. **Figure S3**. Confocal microscope images of NK92-MI cells + PC3 prostate cancer cells + ferumoxytol treated with aPD-L1 or non-aPD-L1 (Target: attached PC3 cells, Effector: NK92-MI cells, E:T = 1:1, Blue: DAPI, and Green: NK92-MI). **Figure S4**. *In vivo* individual tumor growth curve of control group and each treatment group of NK cells, ferroptosis + NK cells, and NK cells + ferroptosis + aPD-L1 (n = 4~ 5 for each group). **Figure S5**. Gating strategies for immune cells

## Data Availability

The datasets used and analysed during the current study are available from the corresponding author on reasonable request.

## Abbreviations

NK: Natural killer
LPO: Lipid peroxide
ROS: Reactive oxygen species
DAMPs: Damage-associated molecular pattern molecules
MHC: Major histocompatibility complex
ULBP: UL16-binding proteins
HMGB1: High mobility group box 1
PD-L1: Programmed death ligand-1
aPD-L1: Anti-programmed death ligand-1
