# Supplementary material for: Enhanced natural killer cell anti-tumor activity with nanoparticles mediated ferroptosis and potential therapeutic application in prostate cancer
Source: J Nanobiotechnology. 2022 Sep 29;20:428. doi: 10.1186/s12951-022-01635-y (PMC9523925; doi:10.1186/s12951-022-01635-y)
Supplement: Supplementary file 1 — Additional file 1: Figure S1. Ferumoxytol concentration dependent Cell viability changes of PC3 and NK92-MI. Ferumoxytol (0–160 μg/mL concentration) was added to each well and incubated for 72 h. Then, CCK-8 reagents were treated by the manufacturer’s instructions. Figure S2. Western blot analysis of PC-3 cells treated with various amounts of ferumoxytol (Fer (0 μg), Fer (50 μg), Fer (100 μg), and Fer (200 μg)) and quantification of relative bands of western blotting. Figure S3. Confocal microscope images of NK92-MI cells + PC3 prostate cancer cells + ferumoxytol treated with aPD-L1 or non-aPD-L1 (Target: attached PC3 cells, Effector: NK92-MI cells, E:T = 1:1, Blue: DAPI, and Green: NK92-MI). Figure S4. In vivo individual tumor growth curve of control group and each treatment group of NK cells, ferroptosis + NK cells, and NK cells + ferroptosis + aPD-L1 (n = 4~ 5 for each group). Figure S5. Gating strategies for immune cells [file 12951_2022_1635_MOESM1_ESM.pdf]

Supplementary Information for

Enhanced Natural Killer Cell Anti-Tumor Activity with Nanoparticles Mediated Ferroptosis and Potential Therapeutic Application in Prostate Cancer

Kwang-Soo Kim[1], Bongseo Choi[1], Hyunjun Choi[1,2], Min Jun Ko[1], Dong-Hwan Kim[3], Dong-Hyun Kim[1,2,4,5]**

[1]Department of Radiology, Feinberg School of Medicine, Northwestern University, Chicago, IL 60611, USA
[2]Department of Biomedical Engineering, University of Illinois, Chicago, IL 60607, USA
[3]School of Chemical Engineering, Sungkyunkwan University (SKKU), Suwon, 16419, Korea
[4]Robert H. Lurie Comprehensive Cancer Center, Northwestern University, Chicago, IL 60611, USA
[5]Department of Biomedical Engineering, McCormick School of Engineering, Northwestern University, Evanston, IL 60208, USA

*Corresponding Authors

Dong-Hwan Kim (dhkim1@skku.edu) and Dong-Hyun Kim (dhkim@northwestern.edu)

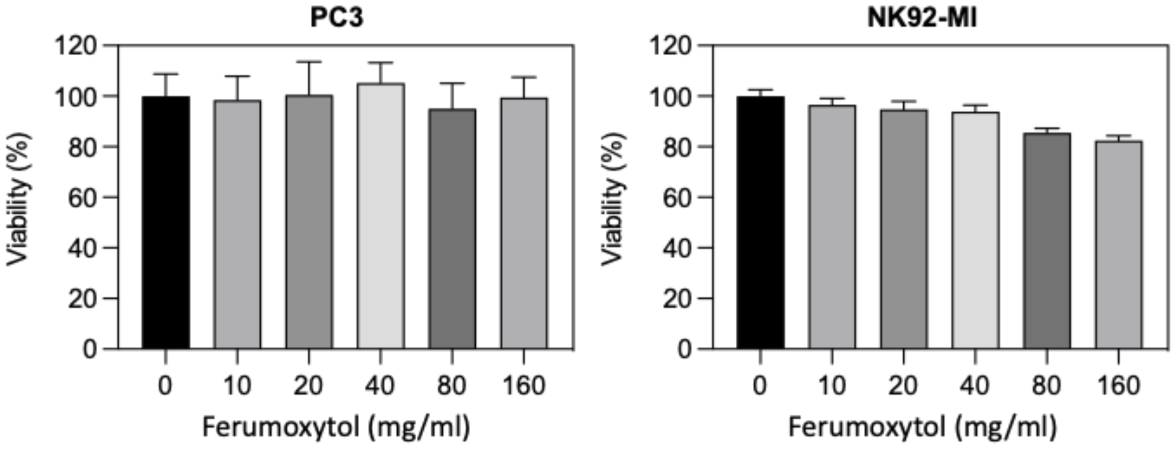

Figure S1. Ferumoxytol concentration dependent Cell viability changes of PC3 and NK92-MI. Ferumoxytol (0 to 160 µg/mL concentration) was added to each well and incubated for 72 h. Then, CCK-8 reagents were treated by the manufacturer's instructions.

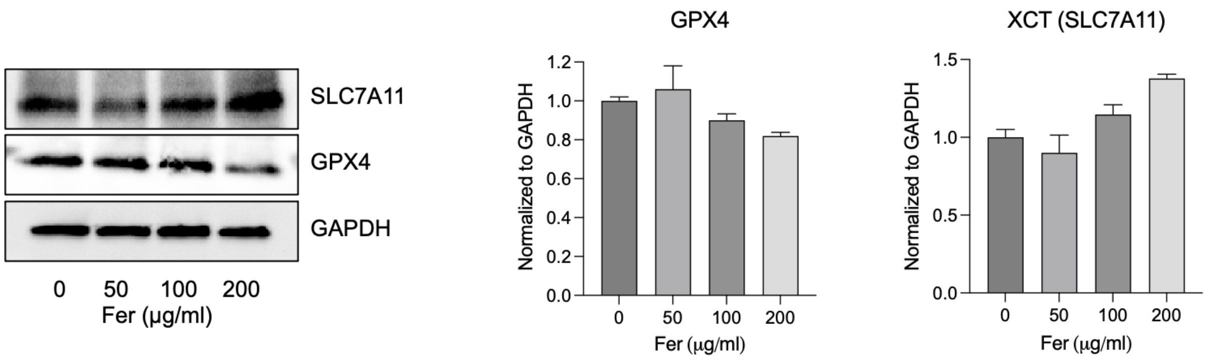

Figure S2. Western blot analysis of PC-3 cells treated with various amounts of ferumoxytol (Fer (0 µg), Fer (50 µg), Fer (100 µg), and Fer (200 µg)) and quantification of relative bands of western blotting. Quantitative comparisons were performed between samples on the same gels/blots. Glutathione peroxidase 4 (GPX4) and solute carrier family 7 member 11 (SLC7A11) are the key regulators in ferroptosis.

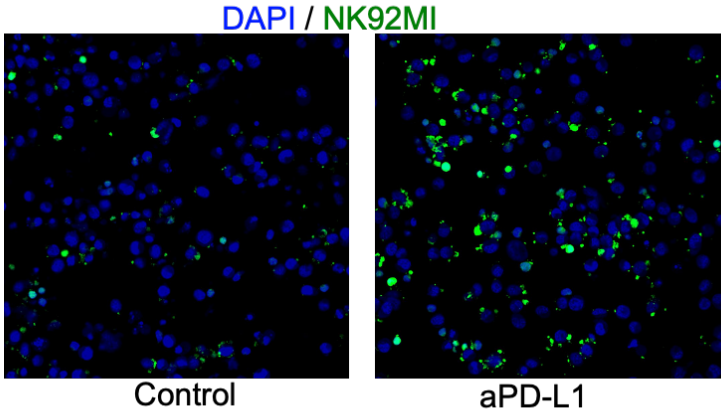

Figure S3. Confocal microscope images of NK92-MI cells + PC3 prostate cancer cells + ferumoxytol treated with aPD-L1 or non-aPD-L1 (Target: attached PC3 cells, Effector: NK92-MI cells, E:T = 1:1, Blue: DAPI, and Green: NK92-MI).

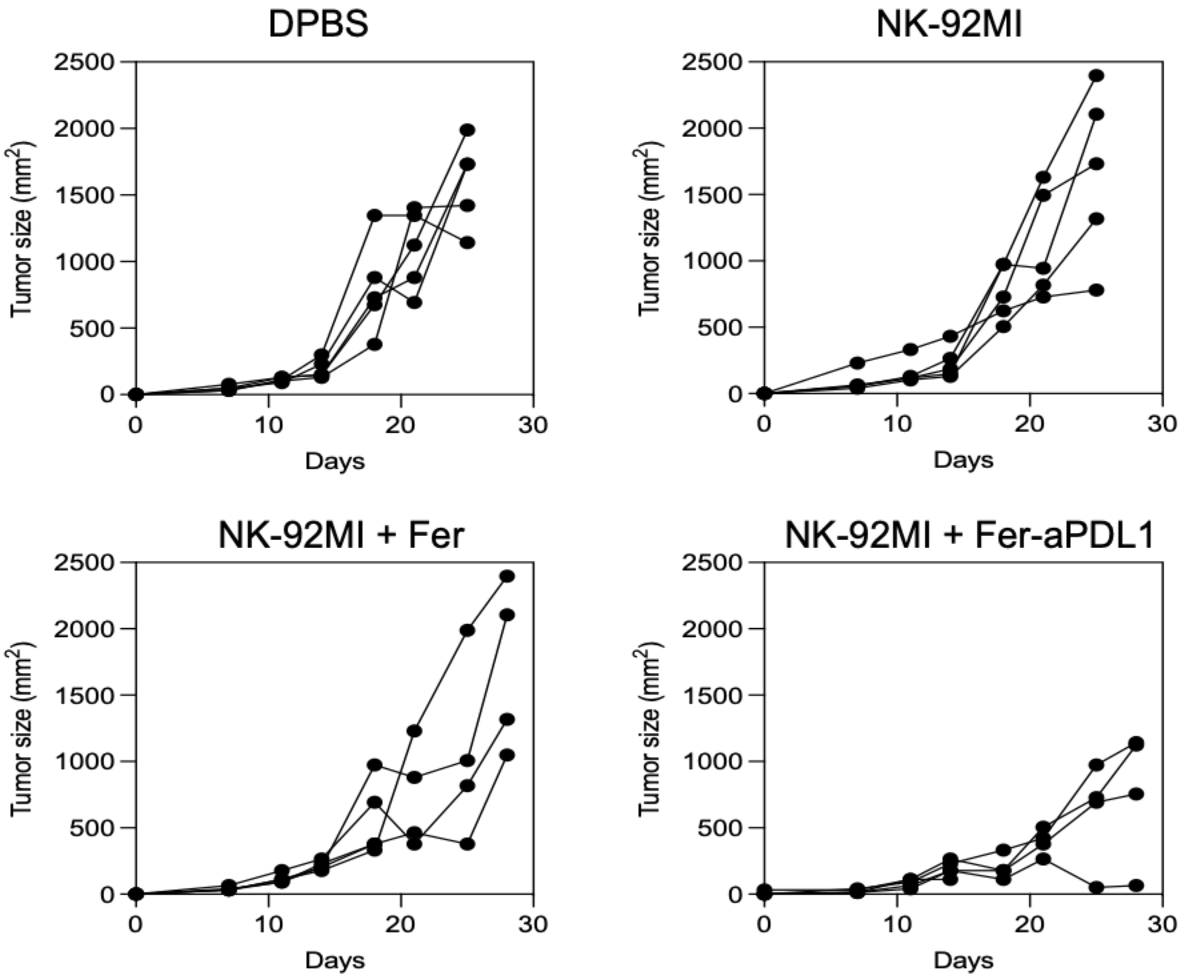

Figure S4. *In vivo* individual tumor growth curve of control group and each treatment group of NK cells, ferroptosis + NK cells, and NK cells + ferroptosis + aPD-L1 (n = 4 ~ 5 for each group).

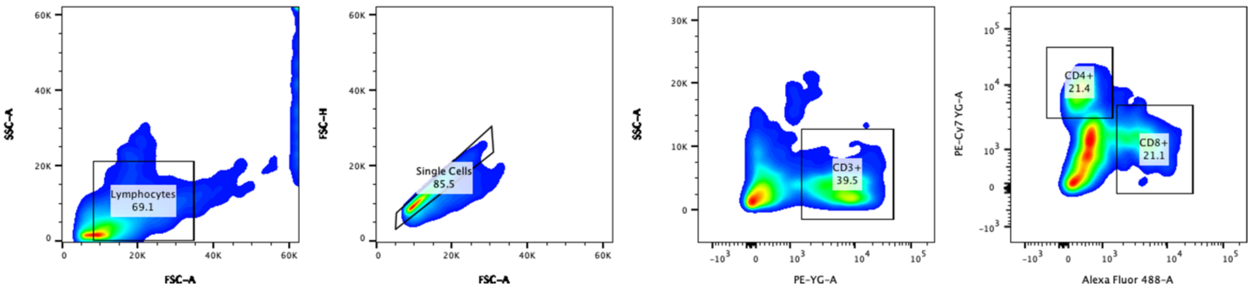

Figure S5. Gating strategies for immune cells. Lymphocytes were gated by forward and side scatter and singlets were gated. T lymphocytes were selected based on CD3 expression. The CD3$^+$ population was further divided into CD4$^+$ and CD8$^+$ T cells based on their expression.
